# Cost-effectiveness of treatments for non-osteoarthritic knee pain conditions: A systematic review

**DOI:** 10.1371/journal.pone.0209240

**Published:** 2018-12-19

**Authors:** Tamana Afzali, Mia Vicki Fangel, Anne Sig Vestergaard, Michael Skovdal Rathleff, Lars Holger Ehlers, Martin Bach Jensen

**Affiliations:** 1 Center for General Practice at Aalborg University, Aalborg, Denmark; 2 Department of Clinical Medicine, Aalborg University, Aalborg, Denmark; 3 Danish Center for Healthcare Improvements, Department of Business and Management, Aalborg University, Aalborg, Denmark; Universitat Bremen, GERMANY

## Abstract

**Introduction:**

Knee pain is common in adolescents and adults and is associated with an increased risk of developing knee osteoarthritis. The aim of this systematic review was to gather and appraise the cost-effectiveness of treatment approaches for non-osteoarthritic knee pain conditions.

**Method:**

A systematic review was conducted according to the PRISMA guidelines and registered on PROSPERO (CRD42016050683). The literature search was done in MEDLINE via PubMed, EMBASE, The Cochrane Library, and the National Health Service Economic Evaluation Database. Study selection was carried out by two independent reviewers and data were extracted using a customized extraction form. Study quality was assessed using the Consensus on Health Economic Criteria list.

**Results:**

Fifteen studies were included. The majority regarded the treatment of anterior cruciate ligament (ACL) injuries, but we also identified studies evaluating other knee pain conditions such as meniscus injuries, cartilage defects, and patellofemoral pain syndrome. Study interventions were categorized as surgical or non-surgical interventions. The surgical interventions included ACL reconstruction, chondrocyte implantation, meniscus scaffold procedure, meniscal allograft transplantation, partial meniscectomy, microfracture, and different types of autografts and allografts. The non-surgical management consisted of physical therapy, rehabilitation, exercise, counselling, bracing, and advice. In general, for ACL injuries surgical management alone or in combination with rehabilitation appeared to be cost-effective. The quality of the economic evaluations was of moderate to high quality.

**Conclusion:**

There was insufficient evidence to give a firm overview of cost-effective interventions for non-osteoarthritic knee pain, but surgical treatment of acute ACL injury appeared cost-effective. There is very little data regarding the cost-effectiveness of non-surgical interventions for non-traumatic knee conditions.

## Introduction

Knee pain is a common symptom in both adolescents and adults[1]. It is a common symptom from around the age of 10 years and by the age of 15 one in every three adolescents will report to have some level of knee pain[2]. The prevalence of knee pain continues to be high all through adulthood[3]. Most commonly, knee pain has a non-traumatic onset[4,5]. In young people the majority will develop knee pain with an insidious onset without a prior trauma while one third will develop pain after a trauma to the knee. The same patterns hold true for adults[6]. Meniscal tears and ligament injuries are some of the most frequent traumatic knee injuries with anterior cruciate ligament (ACL) and medial collateral ligament (MCL) injury being the most common[7]. Furthermore, sports injuries are associated with increased knee pain[8]. The overall burden of sports-related injuries is high with a cost of about EUR 56 m annually for knee/lower leg injuries[8]. The knee has a high weight-loading and changes in the biomechanics of the joint may cause pain and harm the knee[9,10]. Knee pain during adolescence and traumatic injuries such as ACL and meniscal injuries may increase the risk of developing knee osteoarthritis (OA), and subsequent pain and functional impairment[11–13]. These findings constitute a strong rationale behind increasing knee pain treatment efforts and to prevent OA in the longer-term.

The utilization of health care and costs associated with knee pain and knee injuries is substantial. A lifetime cumulative proportion calculation showed about 13% of adults reporting to have seen their general practitioner (GP) and 6.8% was referred to secondary care due to knee pain[14]. In the USA, the incidence rate of cruciate ligament injuries is about 250.000 injuries per year. The associated surgical and the rehabilitative costs of one cruciate ligament injury has been estimated to be about EUR 11.500. This corresponds to a total cost of EUR 2.9 bn per year which signifies the substantial economic burden of knee pain to society[15,16].

The choice of treatment for knee pain depends on the specific diagnosis, the patient, and the available resources. The main treatment options are often classified into: Information/advice, conservative treatment, exercise therapy, medications, surgery, and others (e.g. manipulation and acupuncture[17]. Preventive interventions have shown to be effective in preventing knee pain in young and adult population[18]. However, there is no consensus about the optimal treatment of non-osteoarthritic knee pain[19–21]. Due to an increasing number of treatment approaches available and the global economic burden of knee pain, it is important to increase the knowledge of the cost-effectiveness of the different treatment options for knee pain conditions to enable informed resource utilization. To our knowledge, no systematic review has yet synthesised the evidence on the cost-effectiveness of non-osteoarthritic knee pain management. The aim of this systematic review was to identify, gather, and appraise studies reporting on the cost-effectiveness of different treatment approaches for non-osteoarthritic knee pain conditions in adolescents and adults to identify cost-effective treatments.

## Materials and methods

The present study is based on a systematic review and the reporting follows the PRISMA guidelines[22], See S1 PRISMA. It is prospectively registered in PROSPERO (Registration number: CRD42016050683).

### Study identification

We conducted a systematic search in the following major electronic databases: MEDLINE via PubMed, EMBASE, and The Cochrane Library. Furthermore, a search was conducted for studies related to health economics in the National Health Service Economic Evaluation Database (NHS EED). The search was expanded by reviewing the references of the included studies to identify further relevant publications. The search was not limited to time of publication, but language-restricted to Danish, English, Swedish, and Norwegian.

The search strategy was developed using the Patient, Intervention, Comparator, Outcome (PICO) approach and included thesaurus terms and free terms relating to or describing the condition and health economic outcomes, see S1 Search Strategy. PICO model is a way of defining clinical question in terms of specific patient problem and helps defining the research question. The ‘Patient’ in this review was knee pain conditions and the ‘Outcome’ was cost-effectiveness. Terms related to these words were combined in the search strategy. The initial search was conducted on 28^th^ of November 2016 and was repeated on 28^th^ of March 2017 just before the final analyses to retrieve possible further studies for inclusion. A list of predetermined inclusion and exclusion criteria for study identification is presented in Table 1.

**Table 1:** Inclusion and exclusion criteria.

Inclusion	Exclusion
Full-text paper published in peer-reviewed journals	Not peer-reviewed paper
Model-based and trial-based economic evaluations (e.g. CBA, CUA, CEA, CCA)	Effectiveness studies
Comparison of two or more interventions according to both costs and consequences	Cost studies of single interventions
Adolescents or adults (age 10–65)	Children (age<10) and elderly (65+)
Pharmacological, non-pharmacological, conventional, invasive (surgical), and noninvasive treatments of knee pain in primary and secondary healthcare	Alternative treatment (e.g. healing, mindfulness)
Economic evaluations performed from a societal or/and narrower perspective	Knee pain caused by arthritis or/and osteoarthritis

CBA: cost-benefit analysis, CCA: cost-consequence analysis, CEA: cost-effectiveness, CUA: cost-utility analysis

### Study selection and data extraction

Study selection was carried out through two stages by two independent reviewers (TA and MVF). In the first stage, clearly irrelevant publications were excluded by screening the titles and abstracts. Publications were included in the second stage if the studies met the inclusion criteria from the first stage as well as studies for which exclusion or inclusion could not be made based on title and abstract alone. The full text of potentially eligible studies was retrieved and assessed for eligibility by two independent reviewers (TA and MVF). Any disagreement between the reviewers about the eligibility of a study was resolved through discussion with a third reviewer (MBJ).

The data were extracted by two independent reviewers (TA and MVF) using a standardized data extraction form. See S1 Extraction form. The extracted data were compared for consistency, which was resolved through discussion and assessment of the original paper. Information about study design, participants and intervention characteristics, methods, and outcome measures was extracted.

### Quality assessment and data synthesis

The Consensus Health Economic Criteria (CHEC) list was applied to assess the methodological quality of the economic evaluations by two independent reviewers (TA and ASV)[23], See S1 CHEC list. The CHEC list is composed of 19 questions with each question assigned with either a ‘yes’ or ‘no’, and is recommended for assessing the quality of economic evaluations[23]. The two reviewers confirmed or disconfirmed compliance with each of the assessment questions based on subjective evaluation of the papers. Any disagreement was resolved through discussion and a third review member (LHE) was involved if disagreement remained. S1 Appendix shows the items of the list along with an explanation of how the items were interpreted for application for the included studies. As the checklist does not provide summary scores, score limits were defined by the authors. If a study received ‘yes’ score higher than 75% of all items, it was defined as a high-quality economic evaluation. Studies that scored higher than 50% up to 75% were considered moderate quality evaluations. If studies scored lower than 50%, they were considered low quality evaluations. Furthermore, the different items of the CHEC list were weighted equally in the scoring algorithm. A narrative synthesis was used to analyse, summarize, and present the information provided in the included studies. The collected data was synthesized using a mapping strategy to provide an overview of the economic evidence of surgical interventions versus non-surgical interventions. When comparison was written in the form A vs B, A was considered the intervention and B was considered control. The data was analysed and combined according to the type of knee pain conditions, interventions and/or type of outcome measure. The type of modelling was assessed by finding the method used, e.g. Markov models or decision tree. For cost-effectiveness of interventions, authors conclusions were reported as the different economic evaluations complied with different criteria for cost-effectiveness.

Furthermore, the costs reported in the original studies were converted to 2016 EUR to account for differential timing and different currencies. Figures was converted using the country’s consumer price index from 1^st^ January of the cost year to 1^st^ January of 2016[24]. The costs were eventually converted to EUR using the exchange rates of January 2016[25].

## Results

**Fig 1:**
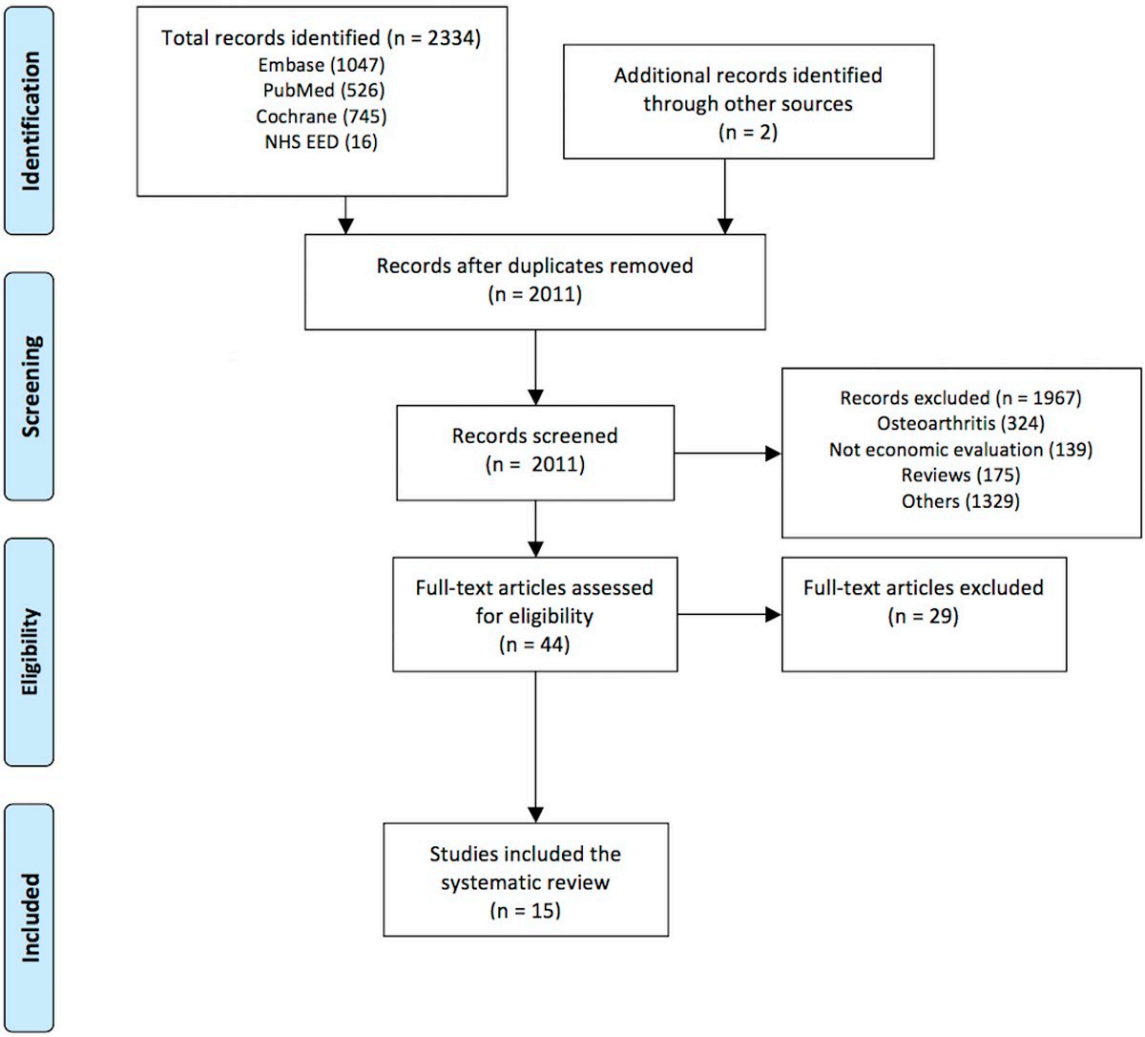
Preferred Reporting Items for Systematic Reviews and Meta-Analyses (PRISMA) flowchart. Numbers in parenthesis in the box indicate the number of studies after each phase. Number in parenthesis in the lowermost box indicates the number of studies included in the review. Fifteen papers were retrained as they met the inclusion and exclusion criteria[26–40].

The initial search resulted in 2334 unique records of which 638 records were excluded because they concerned osteoarthritic knee pain patients, were not economic evaluations, and included reviews. Furthermore, 1329 records were excluded as they did not concern knee pain, were conference abstracts, or the articles did not meet the inclusion criteria. This resulted in 44 records of which 29 were excluded due to the fact that they did concern osteoarthritic knee pain patients, the economic evaluations did not have a comparison or did not include effect in the economic evaluations or included prevention/screening programs. This resulted in 14 studies eligible for inclusion with one additional study found through final search. The present systematic review included 15 studies[26–40], see Fig 1.

### Overview of the identified studies

An overview of the studies is given in Table 2. The studies were conducted from 1999 to 2017 in seven different countries in Europe and the USA and included young and adult patient populations (>16 years of age). The studies evaluated the following pain conditions: ACL injuries, chondral defects, traumatic meniscus injuries, and patellofemoral pain syndrome (PFPS). Of the included studies, seven of the studies compared ACL reconstruction of the knee to different initial non-surgical management forms, including counselling, bracing, and physical therapy, or conservative management with optional surgery[26–32]. Seven studies compared one surgical management to another surgical management. The surgical management forms included ACL reconstruction, chondrocyte implantation, meniscus scaffold procedure, meniscal allograft transplantation, partial meniscectomy, microfracture, and different types of auto- and allografts[33–39]. Only one study assessed the cost-effectiveness of a non-surgical management versus a non-surgical management. The study compared physical therapy to usual care consisting of advice and information[40].

**Table 2:** Overview of the identified studies.

Study (Year)	Interventions	Country	Population	Reference
**Surgery compared to non-surgical initial treatment**				
*Anterior cruciate ligament injury*				
Gottlob et al (1999)	ACL reconstruction with a patellar tendon autograft compared to rehabilitation, counselling and functional bracing.	The United States	Young adults in their late teens and 20’s, acute ACL tear.	[26]
Mather III et al (2013)	ACL reconstruction compared to structured rehabilitation plus optional delayed reconstruction.	The United States	General population age 12–45 years, ACL tear.	[27]
Mather III et al (2012)	Early ACL reconstruction compared to rehabilitation.	The United States	General population age 12–45 years, ACL tear.	[28]
Stewart et al (2016)	ACL reconstruction compared to physical therapy.	The United States	Competitive athletes, complete ACL tear.	[29]
Farshad et al (2011)	Surgical ACL reconstruction compared to conservative treatment.	Switzerland	General population, average age of 30–35 years, ACL rupture.	[30]
Kiadaliri et al (2016)	Structured rehabilitation plus early ACL reconstruction compared to structured rehabilitation plus optional delayed ACL reconstruction.	Sweden	Active adults, 18–35 years of age, acute ACL injury.	[31]
Bierbaum et al (2017)	DIS reconstruction compared to wait and see (muscular training plus delayed ACL reconstruction).	Germany	Patients with an isolated rupture of the ACL with or without meniscal injury.	[32]
**Surgery compared to surgery management**				
*Cartilage defects*				
Derrett et al (2005)	Autologous chondrocyte implantation compared to mosaicplasty.	United Kingdom	General population, 16+ years, chondral or osteochondral lesions of >1 cm diameter.	[33]
Elvidge et al (2016)	Characterised Chondrocyte implantation compared to microfracture.	United Kingdom	General population, aged 18–50, cartilage damage.	[34]
Gerlier et al (2010)	ChondroCelect cell therapy compared to microfracture.	Belgium	Adult patients, aged <50 years, symptomatic cartilage lesions of the femoral condyles.	[35]
*Traumatic meniscus injury*				
Rongen et al (2016)	Meniscus scaffold procedure compared to standard meniscectomy	The Netherlands	General population, mean age of 39 years, patients with an acute traumatic or degenerative irreparable medial meniscus injury.	[36]
Ramme et al (2016)	Meniscal allograft transplantation compared to partial meniscectomy.	The United States	Active athletic women, aged 25–30 years old with normal BMI, discoid lateral meniscus tears.	[37]
*Anterior cruciate ligament*				
Paxton et al (2010)	Single-bundle versus double-bundle autograft ACL reconstruction.	The United States	Young healthy person, ACL tear	[38]
Genuario et al (2012)	Bone–patellar tendon–bone autografts, quadrupled hamstring tendon autografts, and allografts compared with each other.	The United States	Subjects in a sports medicine clinic, ACL injury.	[39]
**Exercise compared to usual care**				
*Patellofemoral pain syndrome*				
Tan et al (2010)	Exercise therapy compared to ‘‘usual care”.	The Netherlands	Adolescents and young adults, age 14–40 years, PFPS.	[40]

ACL: anterior cruciate ligament, BMI: body mass index, DIS: dynamic intraligamentary stabilization, PFPS: patellofemoral pain syndrome.

### Overview of the identified economic evaluations

All included studies performed a cost-utility analysis (CUA) having quality-adjusted life years (QALYs) as outcome measurement[26–36,38–40], except for one, which consisted of a cost-effectiveness analysis (CEA) with outcome measured as years to total knee alloplastic (TKA) and the subsequent incremental cost-effectiveness ratio reported as cost per year to arthroplasty gained[37]. Nine of the studies performed model-based economic evaluations with input data from the best evidence available in the literature[26,29,30,32,33,36–39] while the remaining seven performed economic evaluations with data from clinical trials and cohort studies[27,28,31,34,35,40]. The time horizon of the studies varied from one year to lifetime. A societal perspective was adopted in eight of the studies[27–29,31,36,38,39,40] while five studies adopted a healthcare perspective or third payers’ perspective such as the National Health Service (NHS)[30,32,34,35,37]. The remaining two studies did not explicitly report the actual perspective[26,33]. Utility was measured by the EuroQol-5-Dimensions-3-Levels instrument (EQ-5D-3L), The Short Form (SF)-36 questionnaire, and utility measured based on functional activity classes. Discounting was carried out to account for the effect of preferential timing in 11 out of 13 studies that had a follow-up time greater than one year[26–29,31,32,34–38,40]. Two studies did not explicitly mention the approach to discounting of costs and benefits[30,33]. Furthermore, all the studies performed sensitivity analyses to assess the robustness of the models.

The methodological quality of the studies by assessment of the CHEC list varied with nine of the studies rated as “high quality”[27,29,31,34–37,39,40] and the remaining six rated as “moderate quality”[26,28,30,32,33,38]. Most commonly, the inappropriately reported or missing items in the CHEC list was: 1) Time horizon, 2) perspective of the analysis, 3) discussion of the generalizability of the studies, and 4) discussion of ethical and distributional issues. No studies were rated as “low quality”. Characteristics of the economic evaluations including the CHEC score is given in Table 3.

**Fig 2:**
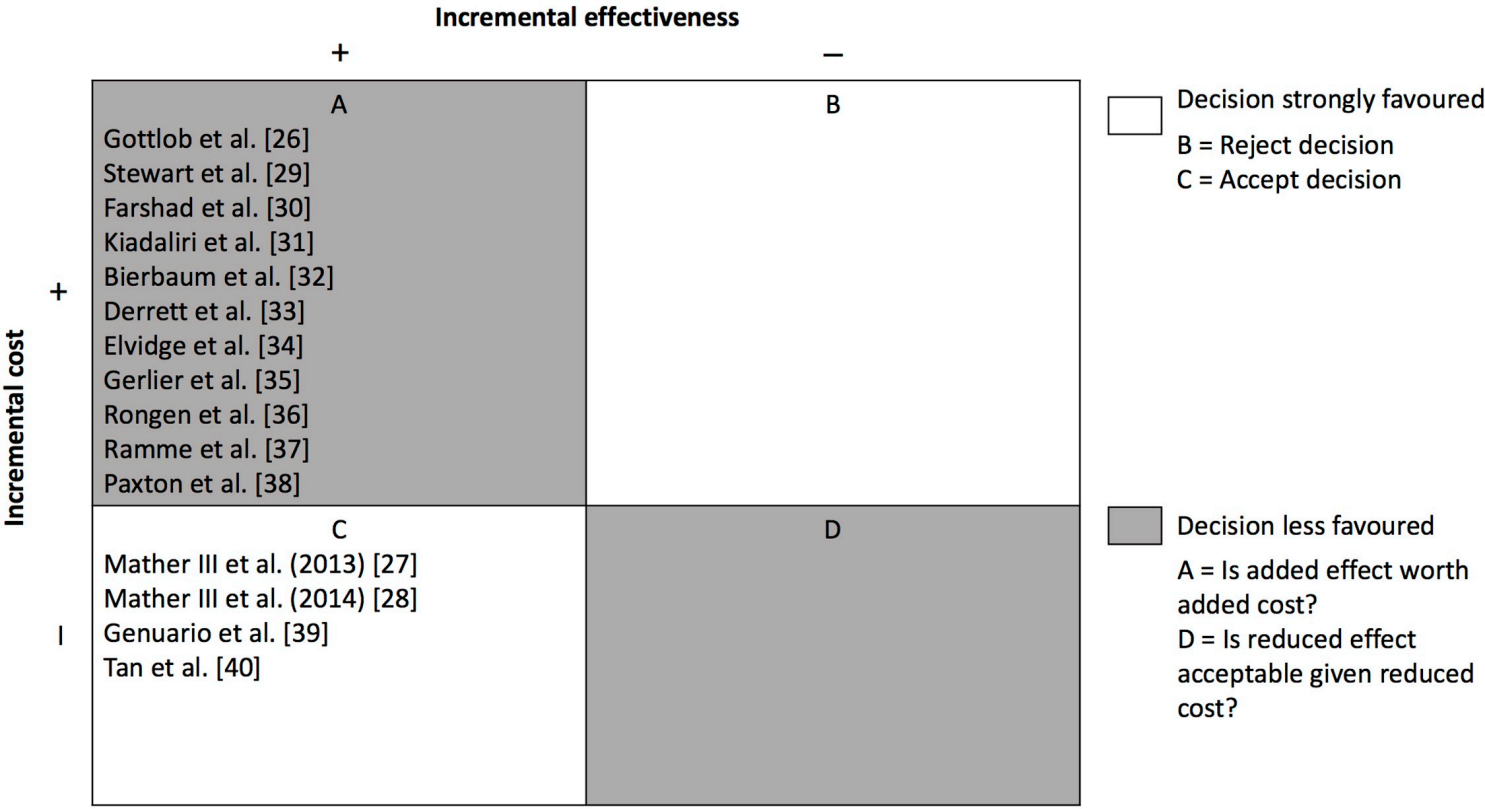
Permutation matrix for possible outcomes of economic evaluations for studies of interventions vs comparator. The letters indicate if the intervention should be accepted, rejected or there is no obvious decision to make. The shading offers a means of more easily identifying the implications for decision making.

Furthermore, a permutation matrix was conducted to summarise the cost-effectiveness of interventions. It is a visual illustration showing permutation that indicate a certain treatments’ incremental cost and incremental effectiveness. Four studies in this review accepted the interventions being assessed with lower costs and better effectiveness associated with the intervention compared to the comparator. Based on the remaining 11 studies, an obvious decision could not be conducted because the interventions had higher costs and lower effectiveness when compared to the comparator. Fig 2 is an illustration of the permutation matrix.

**Table 3:** Characteristics of the economic evaluations.

Study [Reference]	Study type	Time horizon	Perspective	ICER (EUR 2016)	CHEC
**Surgery compared to non-surgical initial treatment**					
*Chronic anterior cruciate ligament injury*					
Gottlob et al [26]	Model-based CUA	Seven years	N/A	7.730 per QALY	53%
Mather III et al [27]	Trial-based CUA	Model 1: Six years Model 2: lifetime	Societal	Model 1: Cost saving of 4.240 and QALY gain of 0.18. Model 2: Cost saving of 47.474 and QALY gain of 0.72.	79%
Mather III et al [28]	Trial-based CUA	Six years	Societal	ER group: 3.713 per QALY DR group: 4.242 per QALY	74%
Stewart et al [29]	Model-based CUA	Six years	Societal	20.778 per QALY	84%
Farshad et al [30]	Model-based CUA	90 months	Third party payers	4.815 per QALY	53%
Kiadaliri et al [31]	Trial-based CUA	Five years	Societal	Early ACL: 25.686 and 3,96 QALY Optional delayed ACL group: 21.060 and 3,83 QALY.	84%
Bierbaum et al [32]	Model-based CUA	Three years	The community of insured citizens.	9,092.66 per QALY	68%
**Surgery compared to surgery management**					
*Cartilage defects*					
Derrett et al [33]	Model-based CUA	Two years	N/A	28.723 per QALY	58%
Elvidge et al [34]	Trial-based CEA	75 years	NHS	28.788 per QALY	89%
Gerlier et al [35]	Trial-based CUA	40 years	Global healthcare payer perspective	18.073 per QALY	84%
*Traumatic meniscus injury*					
Rongen et al [36]	Model-based CUA	Model 1: lifetime Model 2: five years	Societal	297.727 per QALY.	84%
Ramme et al [37]	Model-based CEA	25 years	Healthcare system	771 per-year-gained in time to TKA.	79%
*Anterior cruciate ligament injury*					
Paxton et al [38]	Model-based CUA	12 years postoperatively	Societal	6.420 per QALY	68%
Genuario et al [39]	Model-based CUA	One year	Societal	HS: 5.140 and 0,912 QALY BPTB: 4.917 and 0,966 QALY Allografts: 6.656 and 0,904 QALY	79%
**Exercise compared to usual care**					
*Patellofemoral pain syndrome*					
Tan et al [40]	Trial-based CUA	One year	Societal and healthcare	16.299 saving per QALY gained	79%

ACL: anterior cruciate ligament, BPTB: bone-patellar-tendon-bone, CEA: cost-effectiveness analysis, CUA: cost-utility analysis, HS: hamstring, ICER: incremental cost-effectiveness ratio, N/A: not applicable, TKA: total knee arthroplasty, QALY: quality-adjusted life year.

### Findings of the identified studies

#### Surgical vs non-surgical initial treatment of acute ACL injury

Four studies compared a surgical approach to treatment of ACL injury to a non-surgical management alone. Gottlob et al.[26] compared ACL reconstruction with a patellar tendon autograft to a combination of rehabilitation, counselling, and functional bracing. The surgical treatment resulted in higher costs with increased benefit compared to the non-surgical strategy. This corresponded to an incremental cost-effectiveness ratio (ICER) of EUR 7.730 per QALY, making ACL reconstruction a cost-effective strategy compared to the non-surgical treatment assuming a willingness-to-pay (WTP) of about EUR 18.200 (converted from 1999 CAD to 2016 EUR). Mather et al.[27], Stewart et al.[29], and Farshad et al.[30] compared ACL reconstruction with structured rehabilitation, physical therapy, and conservative treatment, respectively. From a societal perspective, ACL reconstruction provided reduced costs with greater benefit when compared to non-surgical management[28,30]. Although the third-party payer perspective was applied in the study by Farshad et al.[30], the ACL surgery was shown to be cost-effective, despite increased costs (ICER: EUR 4.815 per QALY).

Three studies compared a surgical approach to non-surgical plus optional/delayed surgical management[29,32,33]. The study by Mather et al.[28] compared early ACL reconstruction to rehabilitation plus optional delayed reconstruction. In the six-year time horizon, early reconstruction dominated rehabilitation plus optional delayed reconstruction from a societal perspective meaning that early reconstruction was associated with both reduced cost and increased QALYs. After a five-year follow-up, early reconstruction was associated with a slight increase in costs and increased QALYs from a societal perspective, but the difference from optional delayed reconstruction was not statistically significant[31].

Bierbaum et al.[32] evaluated a new treatment option for ACL reconstruction (dynamic intraligamentary stabilization (DIS)) compared to a ‘wait and see’ approach (rehabilitation with optional delayed ACL surgery) in a German setting. The model identified the DIS strategy to be more effective with 2.34 QALY compared to 2.26 QALY for the ‘wait and see’ approach. Though the costs of the DIS treatment was higher, the study concluded DIS to offer potential benefits to the patient with increased quality of life compared to ‘wait and see’ approach.

#### Surgical vs. surgical treatment of cartilage defects

Derrett et al.[33] found that autologous chondrocyte implantation (ACI) was more expensive and more effective than mosaicplasty for the treatment of chondral defects of the knee cartilage in a young population. The authors concluded that ACI was cost-effective if a WTP threshold of EUR 40.651 (Converted from 2016 GBP) per QALY was applied (ICER: EUR 28.723 per additional QALY). ChondroCyte implantation (CCI) was also examined by Elvidge et al.[34] and Gerlier et al.[35] for treatment of cartilage damage of the knee and symptomatic cartilage lesions of the knee, respectively. In both studies the implantation seemed to be cost-effective with better health outcomes and potentially reduced costs.

#### Surgical vs. surgical treatment of traumatic meniscus injury

Rongen et al.[36] conducted two models comparing a meniscus scaffold procedure with standard treatment defined as partial meniscectomy removing as little of the meniscus as possible. Both models showed ICER values above a WTP threshold of EUR 27.100 (Converted from 2016 GBP) per QALY, indicating that neither of the health technologies would be cost-effective. However, meniscal allografts have shown to reduce pain and improve function in patients with discoid lateral meniscus tears by a model-based CEA conducted by Ramme et al.[37] when compared to partial meniscectomy. Over a 25-year period, a strategy of partial meniscectomy was less expensive compared to using meniscal allograft, but the meniscal allograft strategy postponed TKA by an average of 17.3 years compared to 12.5 years for partial meniscectomy. Thus, although meniscal allograft is costlier, it might be effective in delaying TKA.

#### Surgical vs. surgical treatment of ACL injury

A comparison of three graft types showed that the hamstring autograft was the most effective and the least expensive graft choice when compared to bone-patella-tendon bone autograft and allografts for treatment of ACL injury. Indeed, allografts were the least effective and most costly graft type[39]. A model-based CUA was conducted by Paxton et al. to examine the cost-effectiveness of single-bundle versus double-bundle autograft choices for ACL reconstruction. The double-bundle ACL reconstruction was more expensive and more effective. Paxton et al.[38] concluded that, despite increased upfront costs, the double-bundle reconstruction might be cost-effective.

#### Exercise therapy vs. usual care for treatment of PFPS

The study by Tan et al.[40] is the only economic evaluation, which compared an exercise therapy comprising advice, information and a standardized 6-week exercise program with usual care alone, which was defined as advice and information about the patellofemoral pain syndrome (PFPS). The study was concluded that exercise therapy was a cost-effective treatment approach with reduced costs and increased QALYs compared to usual care for the management of PFPS in adolescents and young adults.

## Discussion

### Identified studies

Due to the diversity of the knee pain conditions and interventions in the identified literature, an overall conclusion regarding cost-effective treatments cannot be made. However, most of the studies regarded ACL injuries, where ACL reconstruction showed to be overall cost-effective. Some studies evaluated different interventions for other knee pain conditions such as cartilage damage and meniscus injuries. Based on the identified literature, there is scarce knowledge about the cost-effectiveness of treatment approaches for both traumatic and, in particular, non-traumatic knee pain conditions.

The present systematic review included a broad search strategy with different knee pain conditions and interventions. Despite that, the systematic search was only able to include 15 studies, which is surprisingly few considering the high prevalence of knee pain and the costs related to it.

Most of the studies are conducted in university hospitals, which could indicate some selection bias as hospitals might have a tradition to conduct more research, hence more economic evaluations due to easier access to resources. Small clinics also provide surgical management more frequently for patients with knee pain, but the patient population is different than patients in a hospital setting. Therefore, it limits the transferability of the study results to other settings and conclusions from the included studies should be drawn with caution.

Choice of treatment for knee pain depends on the condition and the site and severity of the pain, but guidelines recommend a combination of education, advice, and physical therapy as first-line treatment for most knee pain conditions[18–20,41,42]. ACL injury is one of the most common traumatic knee injuries and most of the included studies evaluated the cost-effectiveness of treatments for ACL injuries. Gottlob et al.[26], Stewart et al.[29], Farshad et al.[30], and Kiadaliri et al.[31] found surgical management of ACL injury to be cost-effective when compared to rehabilitation and physical therapy with increased costs, and greater benefits in terms of QALYs. Thus, the cost-effective treatment approach for ACL injury seems to be surgical management in younger patients. Still, it can be discussed if the increased benefits are worth the increased costs. Mather et al.[27] showed similar results when assessing cost-effectiveness of surgical management compared to non-surgical management, but the surgical management was associated with cost savings and QALY gains. This indicated clear dominance of surgical management over non-surgical management and the application of a WTP threshold was not necessary. Exercise therapy and rehabilitation alone was not cost-effectiveness for managing traumatic knee pain conditions like ACL injury in younger patients. When interpreting these results one must bear in mind that the studies have differences in time horizons, perspectives of the economic analyses, and different instruments for measuring QALYs. Furthermore, it is time-consuming to attain full benefit of exercise and rehabilitation interventions and therefore longer time horizons are needed to include all benefits of such non-invasive and non-pharmacological interventions. The study by Tan et al.[40] was the only one assessing the cost-effectiveness of advice and information about non-traumatic knee pain by comparing exercise therapy to advice and information alone. The setting in this study reflects the daily practice in the clinic having patients with PFPS as these patients are given advice and information as well as an integrated exercise program before referral for surgical interventions. The time horizon was only one year, which might not be adequate as the actual clinical benefits of the exercise intervention might be seen with a longer time horizon. However, a longer time horizon would not amend the conclusion of the study as the intervention is associated with lower costs and gain in benefits compared to advice and information alone.

Decisions regarding the cost-effectiveness of the interventions in the studies are drawn based on different sets of rules for decision-making for the various settings and, therefore, judgment on transferability and comparison of the studies must be performed with caution. However, it can be discussed if the included studies can be used as a basis for decision-making as the evidence is scarse and the study has highlighted the lack of knowledge on the cost-effectiveness of interventions aimed at a set of knee pain conditions, including tendinitis, bursitis, dislocation of the knee cap, and MCL.

### Methodological issues and comparability

It is good practice for conducting economic evaluations to include all relevant costs according to the implied perspectives and time horizons[43]. The majority of the included studies adopted a restricted perspective such as the NHS or a healthcare perspective, thus excluding productivity costs. A few studies claimed to apply a societal perspective, but still did not include costs related to lost productivity. Though the application of the different perspectives may be in agreement with local recommendations, it hampers the comparability of the results of the economic evaluations. In general, the studies showed that the interventions had only a modest impact on quality of life, but the additional intervention costs were sufficiently modest to ensure cost-effectiveness. Still, private payments and productivity costs have been shown to constitute a significant share of the total costs of knee pain, for which reason the inclusion and exclusion of such costs might have a sizeable impact on the cost-effectiveness of interventions.

Another factor that limits the accuracy of the reported benefits and economic outcomes is the applied time horizon. Relatively short time horizons are likely to be inadequate in capturing the actual benefits and full extent of the long-term costs. Studies with longer time horizons may to a higher degree include all relevant costs and consequences and potentially attenuate findings, but may potentially also produce conflicting cost-effectiveness results compared to economic evaluations with shorter time horizons. For instance, if a lifetime perspective were adopted in studies evaluating surgical compared to non-surgical strategies for treatment of ACL injuries, it might be concluded that non-surgical interventions are cost-effective because of the low costs of the interventions and the increased benefits achieved over the lifetime course. However, removing studies with short-term time horizons in this review would not affect the overall conclusions.

Conducting economic evaluations based on a single study or decision-analytic modelling both has advantages and disadvantages. The most common type of trial-based economic evaluations are those alongside a RCT. Although in general, some trials select the population randomly from the target population, most of the trials do not sample their population randomly[44,45]. This could violate the external validity of the studies and challenge their generalizability. Nonetheless, having patient-specific data on both costs and benefits is attractive for analyses. It ensures that the accumulation of effects and costs are correlated and enables analyses of the relationship between cost and effect accumulation with adjustment for patients’ characteristics. Although such studies are still undertaken and published, there is a growing use of model-based economic evaluations. Model-based economic evaluations integrate and gathers the best available evidence into a single decision analytical framework to inform on complex and multidimensional medical decision problems. Most of the included studies conducted model-based economic evaluations and might potentially reflect the target population. As model-based economic evaluations are more generalizable it might be more appropriate to conduct such economic evaluations[43].

The health economic impact of medical technologies is often only evaluated in a few settings and as a result decision makers often face considerations of whether cost-effectiveness results from a study conducted in another setting can be transferred to their own jurisdiction or if a new study should be conducted. Due to resource constraints and/or time limits, the second option might not always be possible, leaving the decision makers with existing studies as basis for decision, though they may not reflect the decision makers’ decision situation. Consequently, transferability of foreign study results is increasingly being recognized as an important research field[46]. All the identified studies were conducted in developed countries, including the United States, United Kingdom and European countries. From a clinical perspective, the population in these countries are expected to be somewhat similar and effectiveness of interventions might be transferable. However, the cost-effectiveness results are less likely to be transferable across countries due to variability in the structure and delivery of healthcare systems, differences in costs of healthcare services, and the diversity of usual care[47,48]. For example, it can be discussed if the expected effectiveness of conservative or non-surgical treatment options can be transferred from one country to another as it can vary in different countries and states in the USA. Transferability can also be difficult if the costs associated with an intervention is differs across countries and jurisdictions. Depending on the perspective of an economic evaluation, different costs can be included due to delivery of healthcare services. The lack of transferability of results from one country to another can enhance the conclusions of this review as it cannot be concluded if the intervention is actually cost-effective in a particular country. However, Welte et al. developed a tool for managing the transfer of economic evaluation results which can be used to determine transferability of an economic evaluation to a target country[47]. Therefore, implementing cost-effective interventions is not straightforward based on results of this review. Association between health benefits and risk of interventions is differs in terms of complications, adverse events, instability, and compliance. For instance, there is a highere risk of post-surgical adverse events such as rerupture, or failure of prothesis after surgical management of different non-osteoarthritic knee pain conditions. This can result in lower effect of the treatment and the ratio between costs and effects of the treatment will rise. Therefore, interventions with a highere rates of adverse events tends to be not cost-effective.

### Strengths and limitations

The present review displays particular strengths. The review was prospectively registered in PROSPERO, which seeks to avoid duplication of studies and to reduce the risk of reporting bias by enabling comparison of completed review with what was planned in the protocol. As recommended, key electronic databases were searched by constructing free text and indexing terms. No restrictions were made on the type of economic evaluation or the medical conditions, except for osteoarthritis. Two independent reviewers managed the inclusion process as well as the assessment of the methodological quality of the studies.

Nonetheless, our review poses some limitations. The search strings for the present systematic review were loosely based on the PICO search strategy. However, in the present study the ‘I‘ and the ‘C‘ were deliberately omitted to ensure a more inclusive search, as was in agreement with our research question. A ‘well-built’ PICO question should include all four parts. Including the two parts of the PICO might result in different studies found from the databases.

It is important to assess risk of bias in the included studies in a systematic review, as bias can either overestimate or underestimate the true intervention effect[49]. Economic evaluations need to be methodologically sound so the consequent healthcare decisions are ethically defensible. No validated tool is available to assess risk of bias of economic evaluations, but the CHEC checklist is recommended by the Cochrane to use to assess the methodological quality[49]. The CHEC list was developed by a broad representation of experts on the quality assessment of economic evaluations and they achieved consensus on the list of containing items. However, the list does not analyse the quality of economic evaluations based on modelling studies, which might cause bias as the CHEC list only assess the reporting of the economic evaluation. It is sometimes difficult to ascertain if shortcomings in an economic evaluation is due to poor reporting of economic evaluations or the actual quality of the health economic models was poor. Inadequate reporting could be due to journals accepting a limited number of words in an article thus making extensive explanations almost impossible. Therefore, some of the studies included in the present study that achieved a low score based on the CHEC list might, in fact, be of better methodological quality and vice versa. Heterogeneity in terms of study designs, populations, methods, interventions, and outcome measures meant that a narrative summary was used and a formal meta-analysis of studies was not possible. It can therefore be discussed if such systematic reviews including economic evaluations can have an implication for the decision-makers. Furthermore, the review included complicated interventions (both surgical and non-surgical interventions) which are both difficult to be generalizable and difficult to conduct. This makes it difficult to utilize the effects as well.

### Conclusions

Despite the inclusive search strategy employed in the present systematic review, only 15 economic evaluations were identified for inclusion in the review of economic evaluations on interventions for non-arthritic knee pain conditions. The lack of evidence on the cost-effectiveness of often-used interventions is disquieting considering the continuing need for effective interventions within health sectors under budgetary constraints. The heterogeneity of the included studies limit their comparability and hinder the drawing of overall conclusions on the comparable cost-effectiveness of interventions. The majority of the studies presented economic evaluations of interventions for ACL injuries and were conducted in different countries with different costs included. This might hinder transferability of the study conclusions to other countries and settings. However, in general it appeared that surgical management is cost-effective when compared to non-surgical management. Surgical management was compared to another surgical management for other traumatic knee pain conditions such as knee cartilage defects and meniscus injuries. There is no comparison of cost-effective non-surgical management for these conditions and therefore drawing overall conclusions cannot be drawn. Only one study compared exercise therapy for treatment of non-traumatic condition, PFPS, to usual care. The evidence on cost-effectiveness of treatment approaches for non-traumatic knee pain is inconclusive as there is currently only one study of PFPS and no studies assessing cost-effectiveness of surgical management for this condition.

In general, the methodological quality of the economic evaluations ranged from medium to high quality as assessed by use of the CHEC list. Nonetheless, the transferability of the studies remains unassessed and firm conclusions on the cost-effectiveness of interventions cannot be made. Future studies should focus on assessing cost-effectiveness of both traumatic and non-traumatic knee pain interventions over a lifetime time horizon and apply a societal perspective to include all relevant costs. Furthermore, lack of evidence indicates a need for future studies to assess cost-effectiveness studies to ensure optimal resource use.

## Supporting information

S1 PRISMA The PRISMA list for the systematic review. (PDF)

S1 Search strategy Search strategy from the databases. (PDF)

S1 Extraction form An overall extraction form for all the included studies. (PDF)

S1 CHEC list Quality assessment of the included studies. (XLSX)

S1 Appendix The CHEC list with the interpreted explanations. (PDF)
