# Supplementary material for: Cost-effectiveness of treatments for non-osteoarthritic knee pain conditions: A systematic review
Source: PLoS One. 2018 Dec 19;13(12):e0209240. doi: 10.1371/journal.pone.0209240 (PMC6300294; doi:10.1371/journal.pone.0209240)
Supplement: S1 Search strategy — (PDF) [file pone.0209240.s002.pdf]

S1 Appendix: Search strategy in the applied databases

PubMed:

(((cost-benefit analysis[MeSH Terms]) OR (((((((((((((economic evaluation) OR health economic) OR economic* AND analysis) OR cost effect*) OR cost-effect*) OR cost-benefit*) OR cost-utilit*) OR cost utilit*) OR cost consequence* AND analysis) OR cost-consequence* AND analysis) OR cost minimization analysis) OR cost-minimization analysis) OR economic* AND aspect*) OR health care cost*) OR cost analysis))) AND (((((((patella) OR knee) OR knee joint)) AND ((Pain) OR Arthralgia))) OR ((((patellofemoral pain syndrome) OR ("knee pain"[All Fields] OR "knee ache"[All Fields] OR "knee injury*"[All Fields] OR "anterior cruciate ligament"[MeSH Terms] OR "anterior cruciate ligament injury*"[All Fields] OR "knee fracture*"[All Fields] OR "menisci, tibial"[MeSH Terms] OR "medial meniscus"[All Fields] OR "lateral meniscus injury*"[All Fields]))))))

Embase:

#3	#1 AND #2
#2	**'knee pain'**/de OR **'knee injury'** OR **arthralgia** OR **'anterior cruciate ligament'** OR **'anterior cruciate ligament injury'** OR **'knee meniscus'** OR **'knee meniscus rupture'**
#1	**'economic evaluation'**/de OR **'cost effectiveness analysis'**:lnk OR **'cost utility analysis'** OR **'cost benefit analysis'** OR **'health economics'** OR **'economic aspect'** OR **'cost consequence analysis'** OR **'cost minimization analysis'** OR **'health care cost'**

NHS EED:

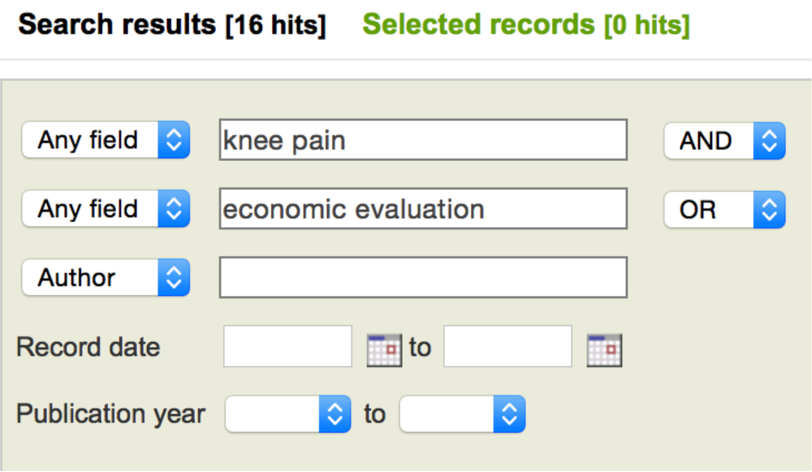

Cochrane:

#1	MeSH descriptor: [Knee] explode all trees
#2	knee pain
#3	patellofemoral pain syndrome
#4	anterior cruciate ligament injury
#5	knee fracture
#6	meniscus tibial
#7	#1 or #2 or #3 or #4 or #5 or #6
#8	MeSH descriptor: [Cost-Benefit Analysis] explode all trees
#9	economic evaluation
#10	cost-effectiveness analysis
#11	cost-utility analysis
#12	cost-minimization analysis
#13	cost-consequence analysis
#14	cost analysis
#15	#8 or #9 or #10 or #11 or #12 or #13 or #14
#16	#7 and #15
