# Supplementary material for: Cost-effectiveness of treatments for non-osteoarthritic knee pain conditions: A systematic review
Source: PLoS One. 2018 Dec 19;13(12):e0209240. doi: 10.1371/journal.pone.0209240 (PMC6300294; doi:10.1371/journal.pone.0209240)
Supplement: S1 Extraction form — (PDF) [file pone.0209240.s003.pdf]

Title/reference: A Cost-Effectiveness Analysis Comparing 3 Anterior Cruciate Ligament Graft Types

**Aim/objective**	CEA is used to analyze the different graft choices in ACL reconstruction to determine the most cost-effective graft.
**Study type**<br>E.g. cost-effectiveness, cost-utility, cost-benefit alongside randomized controlled trial	Cost utility analysis
**Modeling**<br>E.g. decision tree or Markov models	Simplified decision tree
**Duration of study/time horizon/follow-up**	Time horizon was 1 year for a cycle.
**Population, country and perspective of economic evaluation**<br>E.g. Denmark, young adults and societal perspective	Theoretical subjects in a sports medicine clinic with ACL constructions in the Unites States
**Intervention and comparison(s)**	Bone–patellar tendon–bone autografts, quadrupled hamstring tendon autografts, and allografts compared with each other
**Measure of effects**<br>E.g. QALY – measured by EQ-5D or SF-16/SF-56	Utility values using TTO and then estimated to QALY
**Source of cost data**	Costs from multiple sources in the literature. Direct and indirect operative costs, direct postoperative rehabilitation or indirect societal or patient costs
**Currency, cost year and discounting**	US dollars. Not clear what cost year, thus assuming same as the publication year.
**Results – cost calculation**<br>E.g. cost of interventions and effect of interventions, ICER	HS: $5373 and 0,912 QALY, BPTB: $5580 and 0,966 QALY, Allografts: $6958 and 0,904 QALY
**Authors conclusions**	Autograft hamstring ACL reconstruction is the most effective and least expensive<br>graft choice.<br>Both BPTB and allograft reconstructions were dominated by HS
**Other**	

Title/reference: Cost and health status analysis after autologous chondrocyte implantation and mosaicplasty: A retrospective comparison

**Aim/objective**	This paper presents results from a study undertaken by a multidisciplinary team investigating the costs and health status effectiveness of ACI and mosaicplasty.
**Study type**<br>E.g. cost-effectiveness, cost-utility, cost-benefit alongside randomized controlled trial	Cost-utility analysis alongside cross-sectional retrospective cohort study
**Modeling**<br>E.g. decision tree or Markov models	Simple statistical method using Excel and analysis in SPSS.
**Duration of study/time horizon/follow-up**	From first preoperative outpatient appointment to 2 years postoperatively
**Population, country and perspective of economic evaluation**<br>E.g. Denmark, young adults and societal perspective	Subjects with chondral or osteochondral lesions of 1 cm diameter or more and plus 16 years old from the Royal National Orthopaedic Hospital (RNOH) between march 1997 – February 2001 and subjects on ACI waiting list at RNOH between December 2002 and April 2003. United Kingdom
**Intervention and comparison(s)**	Mosaicplasty versus autologous chondrocyte implantation
**Measure of effects**<br>E.g. QALY – measured by EQ-5D or SF-16/SF-56	QALY measured by EQ-5D
**Source of cost data**	RNOH financial services department for 2003-2004. Secondary care cost resource use related to the ACI or mosaicplasty was collected from the patient' electronic medical records at RNOH.
**Currency, cost year and discounting**	British pound 2003/2004<br>No discounting was necessary
**Results – cost calculation**<br>E.g. cost of interventions and effect of interventions, ICER	£10,600 (95% CL:10,036-11,214) and 0,64 QALY for ACI<br>£7,948 (95% CL:6,957-9,243) and 0,47 QALY for Mosaicplasty<br>£23,043/QALY for ACI and £66,043/QALY for mosaicplasty

**Authors conclusions**	Based on costs/QALY, ACI (£23,043 per QALY) was more favorable than mosaicplasty (£66,233 per QALY) with a WTP of £30,000/QALY in both base case and sensitivity analysis.
**Other**	

Title/reference: Cost effectiveness of anterior cruciate ligament reconstruction in young adults

**Aim/objective**	To compared the long-term cost-effectiveness of anterior cruciate ligament reconstruction with nonoperative treatment in isolated acute tears in a young adult population.
**Study type**<br>E.g. cost-effectiveness, cost-utility, cost-benefit alongside randomized controlled trial	Cost-effectiveness analysis Based on outcome data from the past 15 years (before 1999) from six cohort studies.
**Modeling**<br>E.g. decision tree or Markov models	Decision tree
**Duration of study/time horizon/follow-up**	Time horizon of 7 years.
**Population, country and perspective of economic evaluation**<br>E.g. Denmark, young adults and societal perspective	Assumed young adults in their late teens and 20's. Assumed to be in a state of normal health or functional activity level V before their ACL injury.
**Intervention and comparison(s)**	Ligament reconstruction with a patellar tendon autograft compared to rehabilitation, counseling and functional bracing.
**Measure of effects**<br>E.g. QALY – measured by EQ-5D or SF-16/SF-56	QALY measured by direct utility questionnaire based on 4 hypothetical scenarios representing 4 activity classes. Utility values assigned to functional activity 0 to V. mean utility for each functional class were calculated.

**Source of cost data**	Senior authors affiliated outpatient clinic and hospital and compiled data available from orthopedic literature.
**Currency, cost year and discounting**	1999 US dollars, costs and benefits was discounted at a rate of 3% per year.
**Results – cost calculation**<br>E.g. cost of interventions and effect of interventions, ICER	The utilities were 0.86 with costs of $11.768 for operative treatment and 0.57 with $2333 for nonoperative treatment. Marginal cost $9435<br>Marginal effectiveness 1.61 QALY<br>ICER showed $5857 per QALY.
**Authors conclusions**	Concludes that ACL reconstruction is a cost-effective method of treatment for acute tears in properly selected young adults.
**Other**	

Title/reference: Cost Effectiveness of Characterised Chondrocyte Implantation for Treatment of Cartilage Defects of the Knee in the UK

**Aim/objective**	Estimate the cost-effectiveness of ChondroCelect (CC) compared to microfracture in the UK National Health Service.
**Study type**<br>E.g. cost-effectiveness, cost-utility, cost-benefit alongside randomized controlled trial	Cost-utility analysis alongside a randomized controlled trial.
**Modeling**<br>E.g. decision tree or Markov models	Decision tree
**Duration of study/time horizon/follow-up**	Time horizon of 75 years with a cycle length of one month.

**Population, country and perspective of economic evaluation**<br>E.g. Denmark, young adults and societal perspective	128 UK patients aged 18-50 years.<br>NHS perspective
**Intervention and comparison(s)**	Characterised Chondrocyte implantation (CC) compared to microfracture
**Measure of effects**<br>E.g. QALY – measured by EQ-5D or SF-16/SF-56	QALY measured by SF-36 mapped to EQ-5D
**Source of cost data**	NHS reference costs, the Personal Social Services Research Unit and the NICE appraisal process and published health technology assessment report.
**Currency, cost year and discounting**	British pounds in 2014/2015.<br>Costs and benefits discounted at a rate of 3,5 % annually.
**Results – cost calculation**<br>E.g. cost of interventions and effect of interventions, ICER	Incremental cost for CC was £15.229 per patient with a QALY gain of 0,72. ICER showed £21.245 per additional QALY.
**Authors conclusions**	CCI is likely to provide better quality of life and a reduced need for KR surgery and appears to be cost-effective.
**Other**	Uncertainty due to lack of long-term data on clinical outcomes.

Title/reference: Cost effectiveness of meniscal allograft for torn discoid lateral meniscus in young women

**Aim/objective**	Cost-effectiveness analysis of meniscal allograft transplantation in young women for discoid lateral meniscus tears compared to partial meniscectomy.
**Study type**<br>E.g. cost-effectiveness, cost-utility, cost-benefit alongside randomized controlled trial	Cost-effectiveness analysis
**Modeling**<br>E.g. decision tree or Markov models	Markov model. Probability estimates and event rates were obtained from the scientific literature.

**Duration of study/time horizon/follow-up**	Ran the model for 25 years with a 1-year cycle length
**Population, country and perspective of economic evaluation**<br>E.g. Denmark, young adults and societal perspective	1000 active, athletic women aged 25-30 years old with normal BMI and knee alignment with no preexisting osteochondral defects or concomitant injury/infection. Patient with failed conservative management and no diffuse preexisting osteoarthritic changes, synovial disease or inflammatory arthritis.<br>Health system perspective.
**Intervention and comparison(s)**	Meniscal allograft transplantation compared to partial meniscectomy
**Measure of effects**<br>E.g. QALY – measured by EQ-5D or SF-16/SF-56	Postpone of TKA in years gained and the incremental cost-per-year-to-arthroplasty gained.
**Source of cost data**	2014 CMS Medicare Fee Schedule and the Medicare Inpatient Prospective Payment System
**Currency, cost year and discounting**	US dollars, 2014 and discounting costs and benefits with a discount rate of 3%.
**Results – cost calculation**<br>E.g. cost of interventions and effect of interventions, ICER	Partial meniscectomy cost $4040 less at $10,430, compared with $14,470 for the meniscal allograft strategy. Partial meniscectomy postponed TKA an average of 12.50 years, compared with 17.30 years for meniscal allograft, a difference of 4.8 years. Meniscal allograft cost $842 per-year- gained in time to TKA.
**Authors conclusions**	Meniscal allografts were more effective and more costly than partial meniscectomy in delaying TKA over the 25 years we evaluated.
**Other**	

Title/reference: Cost-Effectiveness Analysis of Early Reconstruction versus Rehabilitation and Delayed Reconstruction for ACL Tears

**Aim/objective**	Compare the incremental quality-adjusted life years (QALYs) between early reconstruction (ER) versus delayed reconstruction (DR), and second, to compare the direct costs between these two groups from a societal viewpoint. We hypothesized that the ER group would result in both improved QALYs and less direct expenses.
**Study type**<br>E.g. cost-effectiveness, cost-utility, cost-benefit alongside randomized controlled trial	Cost-utility analysis alongside two randomized controlled trial (KANON study and MOON database)
**Modeling**<br>E.g. decision tree or Markov models	Markov cohort decision model
**Duration of study/time horizon/follow-up**	The model was run for 6 years with 1-year cycle length
**Population, country and perspective of economic evaluation**<br>E.g. Denmark, young adults and societal perspective	The MOON cohort consisted of 807 primary ACL tears with a minimum follow-up of 6 years, while the KANON study had 121 patients (n = 62 in ER group; n = 59 in DR group) and societal perspective
**Intervention and comparison(s)**	Early ACL reconstruction compared to rehabilitation plus optional delayed reconstruction
**Measure of effects**<br>E.g. QALY – measured by EQ-5D or SF-16/SF-36	QALY measured by SF-36 with the use of SF-6D
**Source of cost data**	National average Medicare reimbursements for the procedures, Current Procedural Terminology (CPT) codes for professional costs and private insurer from the literature
**Currency, cost year and discounting**	2012 US dollars, costs and benefits were discounted with a discount rate of 3%.
**Results – cost calculation**<br>E.g. cost of interventions and effect of interventions, ICER	The ER group resulted in an average incremental cost of $1,572 *less than* the DR group, while providing an incremental QALY *gain* of 0.28 compared to the DR group. ICER for ER group was $3,881 per QALY versus $4,434 per QALY for the DR group. The DR treatment strategy was dominated by the ER strategy.
**Authors conclusions**	At the mean, early ACLR is slightly less costly and substantially more cost effective for the patient from a healthcare perspective.

**Other**	

Title/reference: Cost-Effectiveness of Anterior Cruciate Ligament Reconstruction - A preliminary comparison of single bundle and double bundle techniques

**Aim/objective**	The purpose of this study was to use the available published literature to perform a preliminary cost-effectiveness analysis of DB ACL reconstruction compared to single-bundle (SB).
**Study type**<br>E.g. cost-effectiveness, cost-utility, cost-benefit alongside randomized controlled trial	Cost-utility analysis
**Modeling**<br>E.g. decision tree or Markov models	Markov model
**Duration of study/time horizon/follow-up**	12 years postoperative period
**Population, country and perspective of economic evaluation**<br>E.g. Denmark, young adults and societal perspective	Otherwise young healthy person with ACL tear<br>Societal perspective
**Intervention and comparison(s)**	Single-bundle versus double-bundle autograft ACL reconstruction
**Measure of effects**<br>E.g. QALY – measured by EQ-5D or SF-16/SF-56	Utilities were based on a survey of athlete and nonathlete college students. Expert opinion was used to correlate the states reported in this previous study to IKDC grades. An IKDC A outcome was assigned a utility of 1.0, IKDC B was assigned a value of 0.70, IKDC C was 0.33, and IKDC D was 0.23. Standard deviations of the IKDC utilities were calculated according to the total number (N = 285) of responders in the previously described study. Converted to EQ-5D.
**Source of cost data**	Costs from the literature and calculated using gross-cost estimation

**Currency, cost year and discounting**	US dollars, 2009 and costs/health benefits were discounted at 3%.
**Results – cost calculation**<br>E.g. cost of interventions and effect of interventions, ICER	ICER of a DB reconstruction compared with an SB reconstruction is $6416 per QALY. In the reference case the DB reconstruction is both more expensive by $3362, but more effective by 0.52 QALY.
**Authors conclusions**	This preliminary analysis based on published clinical results to date shows DB ACL reconstruction may be cost- effective, despite increased upfront cost. These preliminary results also reveal that cost-effectiveness is most sensitive to the proportion of patients experiencing an IKDC A outcome and less sensitive to the rate of revision surgery and the marginal cost of a DB reconstruction.
**Other**	

Title/reference: Cost-utility analysis of dynamic intraligamentary stabilization versus early reconstruction after rupture of the anterior cruciate ligament

**Aim/objective**	Thus, the aim of our study is to analyze the cost-effectiveness of the dynamic intraligamentary stabilization (DIS) technology in comparison to early reconstructive surgery as a benchmark of the current standard of care after the rupture of the anterior cruciate ligament.
**Study type**<br>E.g. cost-effectiveness, cost-utility, cost-benefit alongside randomized controlled trial	Cost-utility analysis with data input from the literature and a prospective open label study comparing DIS and ACLR.
**Modeling**<br>E.g. decision tree or Markov models	Decision tree
**Duration of study/time horizon/follow-up**	Time horizon of 3 years

**Population, country and perspective of economic evaluation**<br>E.g. Denmark, young adults and societal perspective	The target population of our study are patients with an isolated rupture of the anterior cruciate ligament with or without meniscal injury who are eligible for the treatment with the dynamic intraligamentary stabilization system according to the instructions of use. Study setting is the German public healthcare sector with patients covered by the statutory health insurance. As far as surgical procedures are concerned the analysis is limited to the inpatient setting, because only a small number of patients are treated ambulatory.<br>Perspective of the community of insured citizens in Germany.
**Intervention and comparison(s)**	Dynamic intraligamentary stabilization reconstruction compared to early reconstruction surgery.
**Measure of effects**<br>E.g. QALY – measured by EQ-5D or SF-16/SF-56	QALY measured by SF-36 with the use of SF-6D
**Source of cost data**	G-GRG catalogue, official tariff (Lauer Taxe), payer data, literature, and expert opinions when necessary.
**Currency, cost year and discounting**	Euro 2014, costs discounted with a rate of 3 %.
**Results – cost calculation**<br>E.g. cost of interventions and effect of interventions, ICER	The decision tree analysis identified the DIS strategy as the superior one with 2.34 QALY versus 2.26 QALY for the ACLR branch. The higher QALY also came with higher costs of 5,398.05€ for the DIS branch and 4,632.68€ for the ACLR branch respectively. The resulting ICER is 9,092.66€ per QALY.
**Authors conclusions**	Not only does the DIS technology offer such an early intervention with the potential to benefit patients with an ACL rupture in long term, it also delivers a higher hrQoL in the short term at a favorable ICER of 9092.66€ when it is compared to early reconstruction.
**Other**	

Title/reference: Cost-utility of exercise therapy in adolescents and young adults suffering from the patellofemoral pain syndrome

**Aim/objective**	Determine the cost effectiveness of exercise therapy (intervention group) compared with ''usual care'' (control group) in adolescents and young adults dealing with PFPS in primary care.
**Study type**<br>E.g. cost-effectiveness, cost-utility, cost-benefit alongside randomized controlled trial	Cost-utility analysis alongside a randomized clinical trial
**Modeling**<br>E.g. decision tree or Markov models	Statistical analysis
**Duration of study/time horizon/follow-up**	Randomized trial commenced in august 2005 and finished in may 2007. Follow-up of 1 year.
**Population, country and perspective of economic evaluation**<br>E.g. Denmark, young adults and societal perspective	Adolescents and young adults between 14 and 40 years of age presenting with symptoms of PFPS and no history of previous active treatment with exercises within the last 6 months in The Netherlands. Primarily Societal perspective, the healthcare perspective was also appraised.
**Intervention and comparison(s)**	Exercise therapy (intervention group consisted of advice and information on the background of Patellofemoral pain syndrome by a physician and were appointed to a standardized exercise program, supervised by physiotherapists (nine sessions during 6 weeks), with continuation of home exercises) compared with ''usual care'' (control group consisted of only advice and information on the background of PFPS by a physician, similar to the advice given by general practitioners and sport physicians in a normal care situation).
**Measure of effects**<br>E.g. QALY – measured by EQ-5D or SF-16/SF-56	QALY measured by EQ-5D
**Source of cost data**	College Tarieven Gezondheidszorg/Zorgautoriteit in oprichting, 2002), the collective labor agreement of general practitioner care (College Tarieven Gezondheidszorg/Zorgautoriteit in oprichting, 2005) and the number of workable hours per year (Oostenbrink et al., 2004).<br>Costs for individual patients by multiple resource

	use from the literature.
**Currency, cost year and discounting**	2007 Euro and both healthcare and societal perspective.
**Results – cost calculation**<br>E.g. cost of interventions and effect of interventions, ICER	Intervention group had a total annual cost of €1011 and 0,8722 QALY and control group had a total annual cost of €1.166 and 0,8617. A societal ICER of -€14.738 per QALY.
**Authors conclusions**	Exercise therapy appears to be cost effective as compared with ‘‘usual care.’’
**Other**	

Title/reference: No economic benefit of early knee reconstruction over optional delayed reconstruction for ACL tears: registry enriched randomised controlled trial data

**Aim/objective**	To analyze 5-year cost-effectiveness of early versus optional delayed acute anterior cruciate ligament (ACL) reconstruction.
**Study type**<br>E.g. cost-effectiveness, cost-utility, cost-benefit alongside randomized controlled trial	Cost-effectiveness alongside KANON randomized trial.
**Modeling**<br>E.g. decision tree or Markov models	Stochastic cost-effectiveness analysis
**Duration of study/time horizon/follow-up**	5 year follow-up
**Population, country and perspective of economic evaluation**<br>E.g. Denmark, young adults and societal perspective	Active adults, 18–35 years of age, with an acute ACL injury to a previously uninjured knee presented at the departments of orthopaedics at Skåne University Hospital Lund and Helsingborg Hospital, Sweden. Societal perspective
**Intervention and comparison(s)**	Structured rehabilitation plus early ACL reconstruction compared to structured rehabilitation plus optional delayed ACL reconstruction.

**Measure of effects**<br>E.g. QALY – measured by EQ-5D or SF-16/SF-56	QALY measured by SF-36 (SF-6D was measured). A preference-based measure of health was derived from the SF-36 providing a six-dimensional health state classification (SF-6D). SF-6D values (range worst to best as 0.29–1) were plotted against time and quality-adjusted life years (QALYs) were calculated as the area under the curve.
**Source of cost data**	Diagnostic codes (International Statistical Classification of Diseases and Related Health Problems 10th revision (ICD-10)) and the diagnosis related groups (DRGs) in Skåne Healthcare Register (SHR) Sweden for 2011. KPP database for operations and from Södra-regionvårdsnämnden for inpatient and outpatient visits. Dental and Pharmaceutical Benefits Agency in Sweden for medication prices.
**Currency, cost year and discounting**	2011 Swedish SEK and discounted with a rate of 3%.
**Results – cost calculation**<br>E.g. cost of interventions and effect of interventions, ICER	Early ACL reconstruction group was about 236.000 SEK and 3,96 QALY compared with SEK 193.500 and 3,83 QALY in the optional delayed ACL reconstruction group.
**Authors conclusions**	Rehabilitation plus optional delayed ACL reconstruction provides comparable clinical and economical outcomes after 5 years, as does early and universal ACL reconstruction with rehabilitation.
**Other**	

Title/reference: Reconstruction versus conservative treatment after rupture of the anterior cruciate ligament: cost effectiveness analysis

**Aim/objective**	To identify the more cost effective treatment option for ACL ruptures comparing directly both treatment options in the same study population.

**Study type**<br>E.g. cost-effectiveness, cost-utility, cost-benefit alongside randomized controlled trial	Cost-utility analysis alongside a review of the literature to gather data for model construction.
**Modeling**<br>E.g. decision tree or Markov models	Decision tree model
**Duration of study/time horizon/follow-up**	Time horizon of 90 months
**Population, country and perspective of economic evaluation**<br>E.g. Denmark, young adults and societal perspective	Patients at an average age of 30- 35 years from the viewpoint of third party payers in the Swiss setting.
**Intervention and comparison(s)**	Surgical ACL reconstruction compared to conservative treatment.
**Measure of effects**<br>E.g. QALY – measured by EQ-5D or SF-16/SF-56	QALY measured by clinical vignettes matching activity level I to IV and a questionnaire including the Health Utility Index for utility values, IKDC subjective score and Lysholm and Tegner score was created. Answered by 25 orthopedic surgeons.
**Source of cost data**	Department of Orthopedic Surgery Orthopaedic University Hospital.<br>Swiss National Insurance for Accidents
**Currency, cost year and discounting**	USD calculated by conversion of Swiss Francs (CHF) by a factor of 1.15 based on the exchange rate as of May 3, 2010
**Results – cost calculation**<br>E.g. cost of interventions and effect of interventions, ICER	16,038 USD/0.78 QALY for ACL reconstruction and 15,466 USD/0.66 QALY for conservative treatment, implying a cost effectiveness for the two treatments of 20,612 USD/QALY (SD: 1941 USD/QALY) and 23,391 USD/QALY (SD: 5603 USD/QALY), respectively, and an incremental cost effectiveness of 4890 USD/QALY for ACL reconstruction.
**Authors conclusions**	ACL reconstruction is cost effective. However, although the results of this study might contribute to informed decision making for health policymakers, the individual situation of the patient must be respected when suggesting one or the other strategy.
**Other**	

Title/reference: Societal and Economic Effect of Meniscus Scaffold Procedures for Irreparable Meniscus Injuries

**Aim/objective**	Examine the socioeconomic effect of treating patients with irreparable medial meniscus injuries with a meniscus scaffold either to prevent the development of osteoarthritis or as a treatment strategy for chronic patients. Next, for both scenarios, we also examined which were the most influential variables on the cost -effectiveness of meniscus scaffolds to provide means to maximize the monetary value of providing the interventions.
**Study type**<br>E.g. cost-effectiveness, cost-utility, cost-benefit alongside randomized controlled trial	Cost-utility analysis
**Modeling**<br>E.g. decision tree or Markov models	Two Markov models<br>Model 1: lifetime<br>Model 2: 5-years
**Duration of study/time horizon/follow-up**	Markov model two investigated 5 years cost-effectiveness, Markov model one investigated lifetime cost-effectiveness
**Population, country and perspective of economic evaluation**<br>E.g. Denmark, young adults and societal perspective	Patients with an irreparable medial meniscus injury in The Netherlands. Model 1 included all patients who had an irreparable injury to the medial meniscus (mean age, 39 years). Model 2 specifically included chronic patients who had an irreparable injury to the medial meniscus (mean age, 39 years). Societal perspective
**Intervention and comparison(s)**	Standard treatment (arthroscopic partial meniscectomy, removing as little of the meniscus as possible, and joint debridement if indicated) compared to a meniscus scaffold procedure (standard arthroscopic treatment, followed by the placement of a meniscus scaffold, and joint debridement if indicated).
**Measure of effects**<br>E.g. QALY – measured by EQ-5D or SF-16/SF-56	QALY measured by SF-36 from a randomized trial

**Source of cost data**	Standard reimbursement costs within the Radboud University Medical Center<br>Work Loss Data Institute.<br>Study by Hermans et al
**Currency, cost year and discounting**	2016 Euro and 4.0% and 1.5% discount rate for costs and effects, respectively.
**Results – cost calculation**<br>E.g. cost of interventions and effect of interventions, ICER	Model 1: €5991/0.112 QALY with an ICER of 54,463 per QALY.<br>Model 2: €9825/0.033 QALY with an ICER of 297,727 per QALY
**Authors conclusions**	Results of the current health technology assessment suggest that these treatment strategies should currently not be implemented on a large scale in the health system. However, we identified the most influential variables (cost of the scaffold procedure and quality of life before and after the scaffold procedure) that could be addressed to increase the monetary value of meniscus scaffold procedures.
**Other**	

Title/reference: Societal and economic impact of anterior cruciate ligament tears

**Aim/objective**	The purpose of this study was to examine the societal and economic impact of ACL reconstruction compared with structured rehabilitation only.
**Study type**<br>E.g. cost-effectiveness, cost-utility, cost-benefit alongside randomized controlled trial	Cost-utility analysis alongside two randomized trial (KANON study and MOON database)
**Modeling**<br>E.g. decision tree or Markov models	Markov model
**Duration of study/time horizon/follow-up**	Short and intermediate term with 6 years and life-time with cycle length of one year.

**Population, country and perspective of economic evaluation**<br>E.g. Denmark, young adults and societal perspective	Short term: Population in the MOON study (mean age [and standard deviation], 26 ± 11 years) and KANON study.<br><br>Long term: Data from Losina et al.<br><br>USA and evaluated from societal perspective
**Intervention and comparison(s)**	Anterior cruciate ligament reconstruction compared to rehabilitation.
**Measure of effects**<br>E.g. QALY – measured by EQ-5D or SF-16/SF-56	Utilities were derived from the Short Form-36 (SF-36) values from the MOON cohort with use of the SF-6D, which generates utility values from SF-36 values by adding patient preferences. Estimated to QALY
**Source of cost data**	National mean Medicare reimbursements for the procedures in 2012 U.S. dollars
**Currency, cost year and discounting**	2012 US dollars, and costs/utilities discounted at 3% per year.
**Results – cost calculation**<br>E.g. cost of interventions and effect of interventions, ICER	For short term, mean incremental cost for ACL reconstruction was $4503 less than that for rehabilitation, while providing an incremental QALY gain of 0.18 compared with rehabilitation. ACL reconstruction was shown to be a dominant treatment strategy.<br>For long term, ACL reconstruction resulted in mean incremental cost savings of $50,417 compared with rehabilitation, while providing an incremental QALY gain of 0.72 compared with rehabilitation.
**Authors conclusions**	ACL reconstruction was shown to be cost- saving and more effective in both the short to intermediate term and the long term when compared to rehabilitation. Long-term outcomes, although less certain, appeared to only increase the cost savings of the ACL reconstruction strategy.
**Other**	

Title/reference: The cost utility of autologous chondrocytes implantation using ChondroCelect® in symptomatic knee cartilage lesions in Belgium

**Aim/objective**	To assess the cost effectiveness of CC compared with MFX in the repair of symptomatic cartilage lesions of the knee in Belgium using clinical and QOL data from a phase III RCT.
**Study type**<br>E.g. cost-effectiveness, cost-utility, cost-benefit alongside randomized controlled trial	Cost-utility analysis alongside a randomized controlled trial.
**Modeling**<br>E.g. decision tree or Markov models	A decision tree model.
**Duration of study/time horizon/follow-up**	Time horizon of 40 years
**Population, country and perspective of economic evaluation**<br>E.g. Denmark, young adults and societal perspective	Adult patients aged <50 years with symptomatic cartilage lesions of the femoral condyles who had not yet developed OA in Belgium with global healthcare payer perspective (public payer reimbursement plus possible patient co-payment).
**Intervention and comparison(s)**	ChondroCelect cell therapy compared to microfracture.
**Measure of effects**<br>E.g. QALY – measured by EQ-5D or SF-16/SF-56	QALY measured by SF-36.
**Source of cost data**	The website of the Belgian National Institute for Health and Disability Insurance. Governmental website with statistics by All Patients Refined-Diagnosis Related Group (DRG)<br>Belgian pharmacoeconomic guidelines.
**Currency, cost year and discounting**	2010 Euro and discounted at annual rates of 3% (Costs) and 1.5% (Effects).
**Results – cost calculation**<br>E.g. cost of interventions and effect of interventions, ICER	The use of CC generates an additional cost of €20.802 and a gain of 1.282 QALYs over 40 years compared with MFX. The corresponding ICER is €16.229 per QALY gained.
**Authors conclusions**	CC appears to represent a cost-effective strategy even if scenarios departing from the base case occur in the long run
**Other**	

Title/reference: The Cost-Effectiveness of Anterior Cruciate Ligament Reconstruction in Competitive Athletes

**Aim/objective**	To evaluate the cost-effectiveness of the treatment of acute ACL tears with initial surgical reconstruction versus physical therapy in competitive athletes.
**Study type**<br>E.g. cost-effectiveness, cost-utility, cost-benefit alongside randomized controlled trial	Cost-utility analysis with data input from a literature search
**Modeling**<br>E.g. decision tree or Markov models	Markov model
**Duration of study/time horizon/follow-up**	Time horizon of 6 years with cycle length of 3 months
**Population, country and perspective of economic evaluation**<br>E.g. Denmark, young adults and societal perspective	Competitive athletes with a ACL injury in the USA and a societal perspective
**Intervention and comparison(s)**	Anterior cruciate ligament reconstruction compared to physical therapy.
**Measure of effects**<br>E.g. QALY – measured by EQ-5D or SF-16/SF-56	The generic health status was measured using the Short Form–36 Health Survey (SF-36) from published studies of National Collegiate Athletic Association (NCAA) athletes. The SF-36 score was then converted to an EQ-5D value using a published algorithm that has been tested and found to be reliable and accurate.
**Source of cost data**	Hospital costs for ACL reconstruction, treatment of complications, subsequent knee surgery, physical therapy, and outpatient visits were obtained for a large academic medical center in the US from an activity-based cost accounting system (EPSi-Eclipsys). The average days missed from work/school for both treatment groups was based on the clinical experience of the senior author (D.L.). Time lost in transportation and waiting time for appointments were found in the literature. The average hourly wage from the base group was calculated from the US Census for the age group of 15 to 24 years.
**Currency, cost year and discounting**	2015 US dollars, costs and benefits were discounted at a rate of 3 %.

**Results – cost calculation**<br>E.g. cost of interventions and effect of interventions, ICER	For competitive athletes with a new ACL injury, with over 6 years of follow-up, the ACL reconstruction strategy would be expected to result in 4.675 QALYs at an expected cost of $20,298. Physical therapy would cost $11,853 and 4.303 QALY.<br>The ICER of ACL reconstruction was $22,702 per QALY, which is well below the WTP threshold of $50,000 per QALY gained.
**Authors conclusions**	ACL reconstruction is cost-effective in competitive athletes and provides a reference for payers and decision makers.
**Other**	
